# The impact of increasing femoral width on anatomic risk factors for patellar instability in paediatric patients: a retrospective MRI case-control study

**DOI:** 10.1186/s12891-026-09969-6

**Published:** 2026-06-03

**Authors:** René Schroedter, Amir Koutp, Patrick Sadoghi, Bernhard Guggenberger, Martin Svehlik, Sebastian Tschauner, Tanja Kraus

**Affiliations:** 1 Pediatric and Adolescent Unit, Department of Trauma and Orthopedics, Medical University of Graz, Graz, Austria; 2 Department of Trauma and Orthopedics, Medical University of Graz, Auenbruggerplatz 34, Graz, 8036 Austria; 3 Medical University of Graz, Graz, Austria; 4 Division of Paediatric Radiology, Department of Radiology, Medical University of Graz, Graz, Austria

**Keywords:** Knee, Patellofemoral instability, paediatric, Patellofemoral morphology, Growth

## Abstract

**Background:**

Chronological age does not reliably reflect skeletal development in children and adolescents. Anatomical risk factors for non-traumatic lateral patellar dislocation (LPD) may therefore be better interpreted relative to knee size. This study aimed to evaluate the relationship between femoral width and MRI-based anatomical risk factors for PFI in skeletally immature patients with and without a history of patellofemoral instability.

**Methods:**

In this retrospective MRI case–control study, 630 knee MRIs of skeletally immature patients were analysed, including 315 patients with first-time, non-traumatic lateral patella dislocation and 315 age- and sex-matched controls without a history of PFI. Each group comprised 146 males and 169 females, with a mean age of 14.5 years. MRI-based measurements included parameters of trochlear dysplasia, patellar tilt, patellar lateralization, and patellar height. Bicondylar femoral width was measured to normalize anatomical parameters to knee size. Independent t-tests compared group differences, and linear regression analyses assessed associations between femoral width and patellofemoral parameters.

**Results:**

Compared with controls, patients with PFI showed significantly reduced trochlear depth (3.6 ± 0.09 mm vs. 4.5 ± 0.09 mm), lower lateral trochlear inclination (14.4° ± 0.29 vs. 20.8° ± 0.32), greater trochlear sulcus angle (151.0° ± 0.58 vs. 136.5° ± 0.50), lower Laurin angle (− 0.7° ± 0.55 vs. 2.5° ± 0.57), lower Fulkerson angle (2.2° ± 0.55 vs. 8.6° ± 0.57), higher patellar inclination angle (TILT; 21.2° ± 0.53 vs. 18.5° ± 0.55), greater TTTG distance (13.3 ± 0.27 mm vs. 12.0 ± 0.27 mm), and greater TTPCL distance (23.1 ± 0.33 mm vs. 19.4 ± 0.34 mm), as well as a higher Caton–Deschamps Index (1.2 ± 0.01 vs. 1.1 ± 0.01) (all *p* < 0.05). The Patellotrochlear Index did not differ significantly between groups (47.1 ± 1.09 vs. 47.5 ± 1.02; *p* = 0.764). Linear regression analysis demonstrated significant associations between increasing femoral width and several parameters, including trochlear depth, patellar inclination, Fulkerson angle, TTPCL distance, Patellotrochlear Index, and Caton–Deschamps Index in at least one cohort (*p* < 0.05).

**Conclusion:**

Anatomical risk factors for non-traumatic LPD differ significantly between skeletally immature patients with and without a history of patellar dislocation. Several patellofemoral parameters vary with femoral width, suggesting that knee size should be considered when interpreting patellofemoral morphology during skeletal development.

**Supplementary Information:**

The online version contains supplementary material available at 10.1186/s12891-026-09969-6.

## Introduction

Lateral patellar dislocation (LPD) represents an important cause of knee injury in adolescents, with a reported incidence of 6–50 per 100,000 individuals aged 10–17 years [1]. Per definition LPD refers to the acute event of the patella dislocating laterally from the trochlear groove, whereas patellofemoral instability (PFI) describes the broader clinical condition characterized by recurrent dislocation, subluxation, or persistent instability of the patella [2–4]. Atraumatic lateral patellar dislocation predominantly affects young athletes, comprising 88% of cases, and approximately one-third progress to bilateral patellofemoral instability (PFI) with recurrent dislocations. While fewer than 1% of first-time LPDs are due to major trauma, most cases are linked to underlying anatomical risk factors [5, 6]. Treating PFI during growth is challenging, as nearly 50% of affected individuals develop patellofemoral osteoarthritis within 25 years [7].

Several anatomical and biomechanical contributors to atraumatic LPD have been identified, including trochlear dysplasia, patella alta, patellar tilt, lateralization (e.g., increased TTTG/TTPCL distance), and rotational malalignment [2, 6, 8]. High-risk motions like valgus loading during twisting exacerbate instability [2, 4, 9–13]. In response, individualized treatment approaches are emerging, aided by predictive tools such as the Patella Instability Severity Score (PISS) and Patella Instability Probability (PIP) Calculator [14–16].

Chronological age alone may not adequately reflect skeletal development in children and adolescents, as growth patterns and body size vary considerably between individuals of the same age [5, 17–19]. Previous studies have shown that femoral width correlates with overall femur length and body height, suggesting that it may serve as a surrogate marker for knee size during skeletal development [20–22]. Hirschmann et al. [23] have recently advocated for tailored treatment strategies. Therefore, normalizing patellofemoral parameters to knee size may provide a more individualized framework for interpreting anatomical risk factors in growing patients.

Several studies have examined anatomical risk factors for patellofemoral instability and developmental changes in patellofemoral morphology during skeletal growth in relation to chronological age [17, 24–28]. However, the relationship between these parameters and knee size— in particular femoral width—has not been systematically investigated in skeletally immature patients.

Therefore, the aim of this study was to evaluate how MRI-based anatomical risk factors for patellofemoral instability vary in relation to femoral width in skeletally immature patients with and without a history of patellar dislocation.

## Materials and methods

This retrospective study evaluated magnetic resonance imaging (MRI) scans of skeletally immature patients with a history of first-time, non-traumatic lateral patellar dislocation (LPD) at our institution, comparing them with MRIs from age and sex matched peers without a history of PFI. All scans were retrieved from the local PACS (Picture Archiving and Communication System) server. The study received approval from the local institutional review board and was conducted in accordance with the Declaration of Helsinki. Due to its retrospective nature, informed consent was not required.

**Fig. 1:**
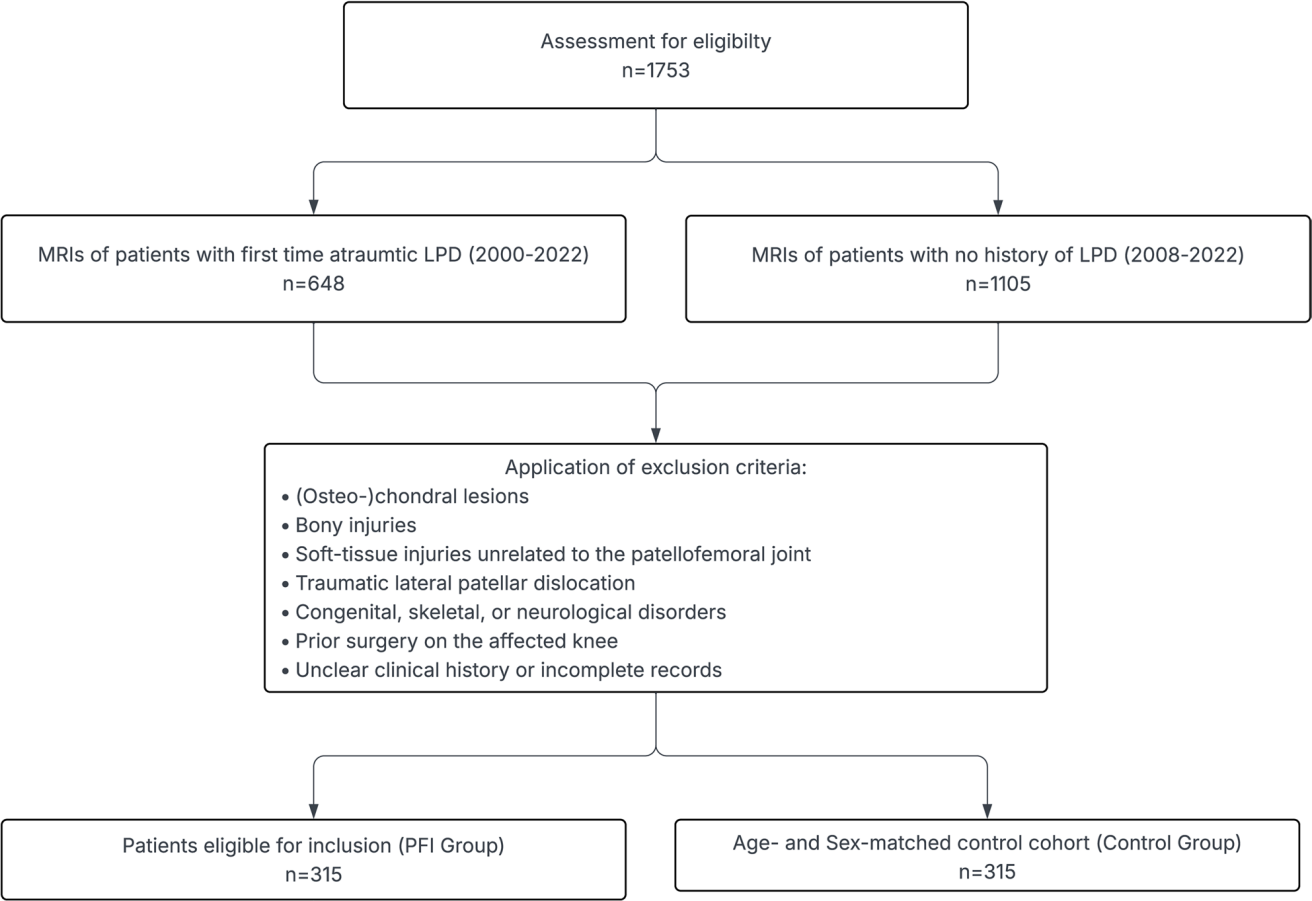
Flow chart of patient screening

Inclusion criteria for the PFI group were patients under 18 years of age who experienced a first-time, non-traumatic lateral patellar dislocation. Non-traumatic (atraumatic) lateral patellar dislocation was defined as a dislocation occurring without direct impact or collision to the patella during the injury mechanism. Exclusion criteria for both groups included an unclear clinical history, incomplete records, osteochondral lesions, other bony injuries (e.g., tibial plateau or femoral condyle fractures), soft tissue injuries unrelated to the patellofemoral joint (e.g., cruciate ligament, meniscal, or collateral ligament injuries), a history of prior surgery on the affected knee as well as traumatic lateral patellar dislocation. Traumatic dislocation was defined as a dislocation associated with direct trauma to the anterior knee or patella. Patients with congenital, skeletal, or neurological disorders known to substantially affect musculoskeletal development or neuromuscular control (e.g., skeletal dysplasia, congenital limb deformities, or neuromuscular disorders such as cerebral palsy) were excluded. Furthermore, patients with hypermobility syndromes (e.g. Ehlers-Danlos-Syndrome or Marfan-Syndrome) were also excluded. Generalized joint hypermobility according to the Beighton-Score was not considered an exclusion criteria unless associated with a documented hypermobility syndrome as well as any congenital or neuromuscular disorder affecting knee morphology. Data collection spanned from 2000 to 2022. A flow chart representing patient screening is shown in Fig. 1.

Out of 648 knee MRI scans from PFI patients, 315 met the inclusion criteria. A control group was formed by randomly selecting an additional 315 age- and sex-matched MRIs from the same institutional database. Age matching was determined based on the patient’s age at the time of the MRI. Propensity score matching was not applied. Anatomical parameters relevant to patellofemoral alignment and instability were measured in both groups, accounting for individual knee size. For PFI group inclusion, patients required at least one documented patellar dislocation, and only the first MRI acquired following diagnosis was analysed.

The control group consisted of age- and sex-matched patients who underwent knee MRI for non-specific knee pain or minor trauma (e.g., sprain or contusion) without any history of patellofemoral instability. Controls with osteochondral fractures, significant ligamentous or bony injuries, or any history of knee surgery were excluded to prevent alteration of anatomical measurements.

### MRI measurements

MRI scans were systematically reviewed to assess anatomical risk factors associated with PFI. Measurements were performed by two board-certified orthopaedic surgeons (> 15 years experience) and two orthopaedic residents (> 5 years experience). Interrater reliability was good to excellent for all measurements (ICCs 0.755–0.976), with the exception of TD (ICC = 0.702), and the CDI (ICC = 0.533), which demonstrated moderate reliability. Intrarater reliability was good to excellent across all measurements (ICCs 0.796–0.984), except for the CDI, which reached only moderate reliability (ICC = 0.664).

Trochlear morphology, a key component of PFI risk, was evaluated using three quantitative metrics: trochlear depth, trochlear sulcus angle, and lateral trochlear inclination angle (Additional file 1: Fig. 6A–C). These measurements have demonstrated clinical utility in identifying trochlear dysplasia [11, 13].

Patellar tilt was assessed using three angular parameterss: trochlear depth, trochlear sulcus angle, and lateral trochlearination angle (TILT) (Additional file 1: Fig. 7) [10]. Patellar lateralization was evaluated using the tibial tuberosity to trochlear groove (TTTG) distance and the tibial tuberosity to posterior cruciate ligament (TTPCL) distance (Additional file 1: Fig. 8) [11, 12].

Sagittal patellofemoral alignment, known to influence dynamic patellar tracking and overall joint stability, was assessed using the Patellotrochlear Index (PTI) (Additional file 1: Fig. 9) and the Caton-Deschamps Index (CDI) (Additional file 1: Fig. 10). Both indices are widely recognized for their validity in skeletally immature populations [4, 9].

To normalize measurements relative to patient-specific knee size, bicondylar femoral width was measured at the furthest points of the subchondral bone of the medial and lateral femoral epicondyles (Additional file 1: Fig. 6D). This adjustment was designed to provide an individualized framework for interpreting morphometric values in growing children and adolescents. All figures referenced in the methods are included in the Additional file 1.

Image analysis and measurements were conducted using the MicroDicom DICOM viewer (MicroDicom Ltd., Sofia, Bulgaria). All measurements were conducted on T2 Turbo-Spin-Echo (TSE) and fatty saturated (FS) sequences with a slice thickness of 3 millimeters. Furthermore, transversal slicing was used for the measurements of trochlea dysplasia parameters, patellar tilt parameters, lateralization of the patella and femoral width. Measurement on sagittal images was used for sagittal alignment of the patella. Extracted demographic variables included age at the time of MRI, date of scan, and laterality of the examined knee.

### Statistical analysis

All statistical analyses were performed using SPSS Version 28.0 (IBM, Armonk, New York, USA). Q-Q plots were used to confirm normal distribution of all variables. Due to the retrospective study design, no a priori sample size calculation was performed. The sample size was determined by the number of eligible MRI examinations available in the institutional database during the study period. All cases were included according to the predefined inclusion and exclusion criteria to maximize statistical power. Group matching was evaluated using independent sample t-tests and standardized mean differences for age and femoral width.

To identify statistically significant differences in mean anatomical values between the PFI and control groups, independent t-tests were employed. Pearson’s correlation coefficient was used to determine the relationship between patient age and femoral width across the full cohort. Results for the independent t-test as well as the Pearson´s correlation coefficient were considered statistically significant at *p* < 0.05. Where applicable, results are reported with corresponding mean difference and 95% confidence intervals (CI95%) to improve the interpretability of the statistical estimates. Linear regression models were created for both the PFI and control groups to investigate the relationship between knee size (as a continuous independent variable) and all patellofemoral geometric measurements (as continuous dependent variables). Additionally, a one-way analysis of variance (ANOVA) was performed within each group to detect significant differences in measured parameters across knee size categories. A p-value < 0.05 was considered statistically significant for all comparisons.

## Results

### Patient demographics

A total of 315 knees with a confirmed history of first-time, non-traumatic lateral patellar dislocation (LPD) were analysed. The age- and sex-matched control group also included 315 knees, with both groups comprising 146 males and 169 females. As detailed in Table 1, there were no statistically significant differences in age or femoral width between the groups.

**Table 1:** Patient demographics

	PFI	CONTROL	Standardized mean difference
Age (y; mean)	14.5 (7.9–17.9)	14.5 (7.9–17.9)	< 0.01
Femoral width (mm)	77.8 (59.5–94.4)	77.7 (57.6–95.7)	< 0.01
Sex	146 male	146 male	
	169 female	169 female	
Laterality	179 left 136 right	160 left 155 right	

Shows the demographic data of the PFI group and the control group without any history of PFI (y=years; mm=millimetres)

Pearson’s correlation analysis using age and femoral width as continuous variables demonstrated a weak correlation between age and femoral width in the full cohort (*R* = 0.270; *p* < 0.001). When comparing mean values, all patellofemoral parameters except the Patellotrochlear Index (PTI) differed significantly between groups (*p* < 0.05), with PTI showing no significant difference (*p* = 0.764). Table 2.

**Table 2:** Comparison of mean values PFI vs. control group

Measurement	PFI (Mean ± SEM)	CONTROL (Mean ± SEM)	Mean difference (CI95%)	*p*-value
TD	3.6 ± 0.09	4.5 ± 0.09	0.85 (0.61–1.10)	< 0.001*
LTIA	14.4 ± 0.29	20.8 ± 0.32	6.40 (5.55–7.25)	< 0.001*
TSA	151.0 ± 0.58	136.5 ± 0.50	14.49 (12.97-16.00)	< 0.001*
LAUR	-0.7 ± 0.55	2.5 ± 0.57	3.25 (1.70–4.80)	< 0.001*
FULK	2.2 ± 0.55	8.6 ± 0.57	6.38 (4.84–7.93)	< 0.001*
TILT	21.2 ± 0.53	18.5 ± 0.55	2.66 (1.16–4.16)	< 0.001*
TTTG	13.3 ± 0.27	12.0 ± 0.27	1.24 (0.50–1.98)	0.001*
TTPCL	23.1 ± 0.33	19.4 ± 0.34	3.69 (2.76–4.62)	< 0.001*
PTI	47.1 ± 1.09	47.5 ± 1.02	0.45 (-2,48-3.38)	0.764
CDI	1.2 ± 0.01	1.1 ± 0.01	0.11 (0.07–0.14)	< 0.001*

Shows mean, standard error of the mean (SEM), mean difference with 95% confidence intervals (CI95%) as well as the results of independent t-test analysis for a difference between the groups

*PFI *Patellofemoral instability group, *CONTROL *Control group, *SEM *Standard error of the mean, *TD *Trochlea depth, *LTIA *Lateral trochlea inclination angle, *TSA *Trochlea sulcus angle, *LAUR *Angle of Laurin, *FULK *Angle of Fulkerson, *TILT *Patellar inclination angle, *TTTG *Tibial tubercle to trochlea groove distance, *TTPCL *Tibial tubercle to posterior cruciate ligament distance, *PTI * Patellotrochlear-Index, *CDI *Caton-Dechamps-Index

*p-values that reached statistical significance (*p* < 0.05)

All statistically significant findings discussed below are normalized to a 10 mm increase in bicondylar femoral width.

### Trochlear dysplasia

**Fig. 2:**
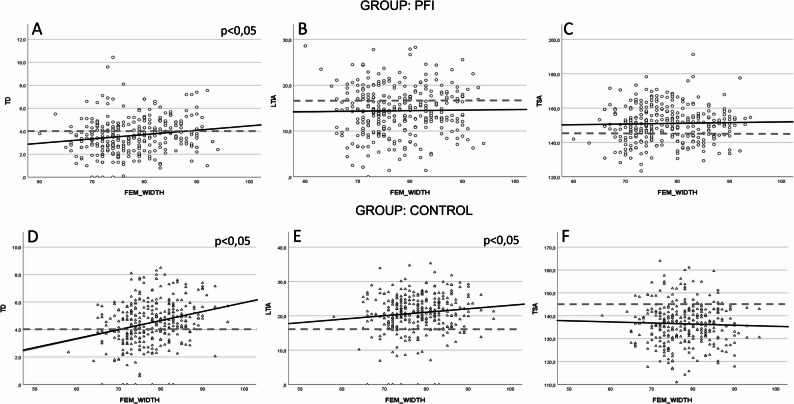
Illustrates the changes for trochlear dysplasia, represented by trochlear depth [TD] (**A**,**D**), lateral trochlear inclination angle [LTIA] (**B,E**) and the trochlear sulcus angle [TSA] (**C**,**F**) in relation to femoral width for both the PFI and control groups. The dashed lines are marking approximate cut offs for abnormal values in literature on adult PFI for TD (<4 mm), LTIA (<17°) and TSA (<145°)

Trochlear depth increased in both groups with rising femoral width—by 0.4 mm in the PFI group (*p* = 0.003; R²=0.03) and 0.7 mm in controls (*p* < 0.001; R²=0.07) (Fig. 2A, D). The trochlear sulcus angle remained unchanged in both groups (Fig. 2C, F). Lateral trochlear inclination angle (LTIA) increased by 1° in controls (*p* = 0.047; R²=0.01), with no change in the PFI group (Fig. 2B, E).

### Patellar tilt

**Fig. 3:**
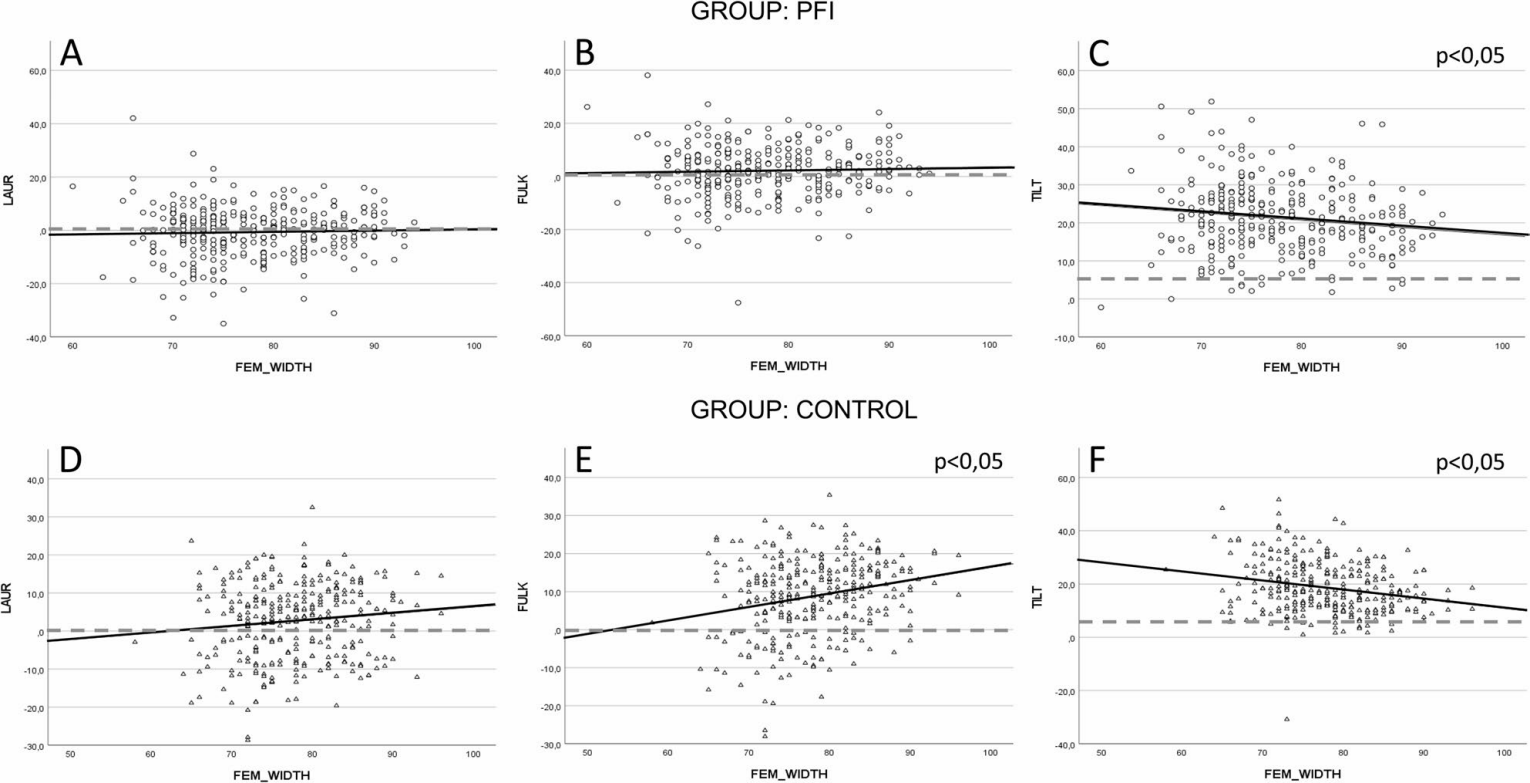
Illustrates the changes for patellar tilt, represented by Angle of Laurin [LAUR] (**A**, **D**), the Angle of Fulkerson [FULK] (**B**, **E**) and the patellar inclination angle [TILT] (**C**, **F**) in relation to femoral width for both the PFI and control groups. The dashed lines are marking approximate cut offs for abnormal values in literature on adult PFI for LAUR (<0°), FULK (<0°) and TILT (>5°)

In controls, the Fulkerson angle increased by 3.5° (*p* < 0.001; R²=0.05), while no significant change was found in the PFI group (Fig. 3B, E). The Laurin angle remained stable in both cohorts (Fig. 3A, D). The patellar inclination angle (TILT) decreased significantly by 1.9° in the PFI group (*p* = 0.013; R²=0.02) and 3.4° in controls (*p* < 0.001; R²=0.05) (Fig. 3C, F).

### Axial patellar positioning

**Fig. 4:**
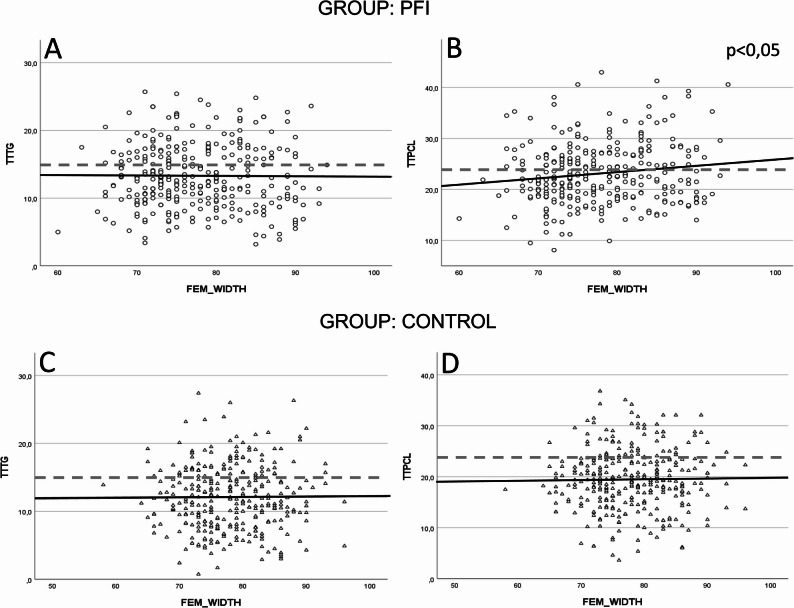
Illustrates the changes for patellar lateralization, represented by tibial tubercle to trochlear groove distance [TTTG] (**A**, **C**) and tibial tubercle to posterior cruciate ligament distance [TTPCL] (**B**, **D**) in relation to femoral width for both the PFI and control groups. The dashed lines are marking approximate cut offs for abnormal values in literature on adult PFI for TTTG (>15 mm) and TTPCL (>24 mm)

No significant correlation was observed between femoral width and TTTG or TTPCL distances in controls. However, the PFI group showed a 1.2 mm increase in TTPCL (*p* = 0.014; R²=0.01) (Fig. 4A–D).

### Sagittal patellofemoral alignment

**Fig. 5:**
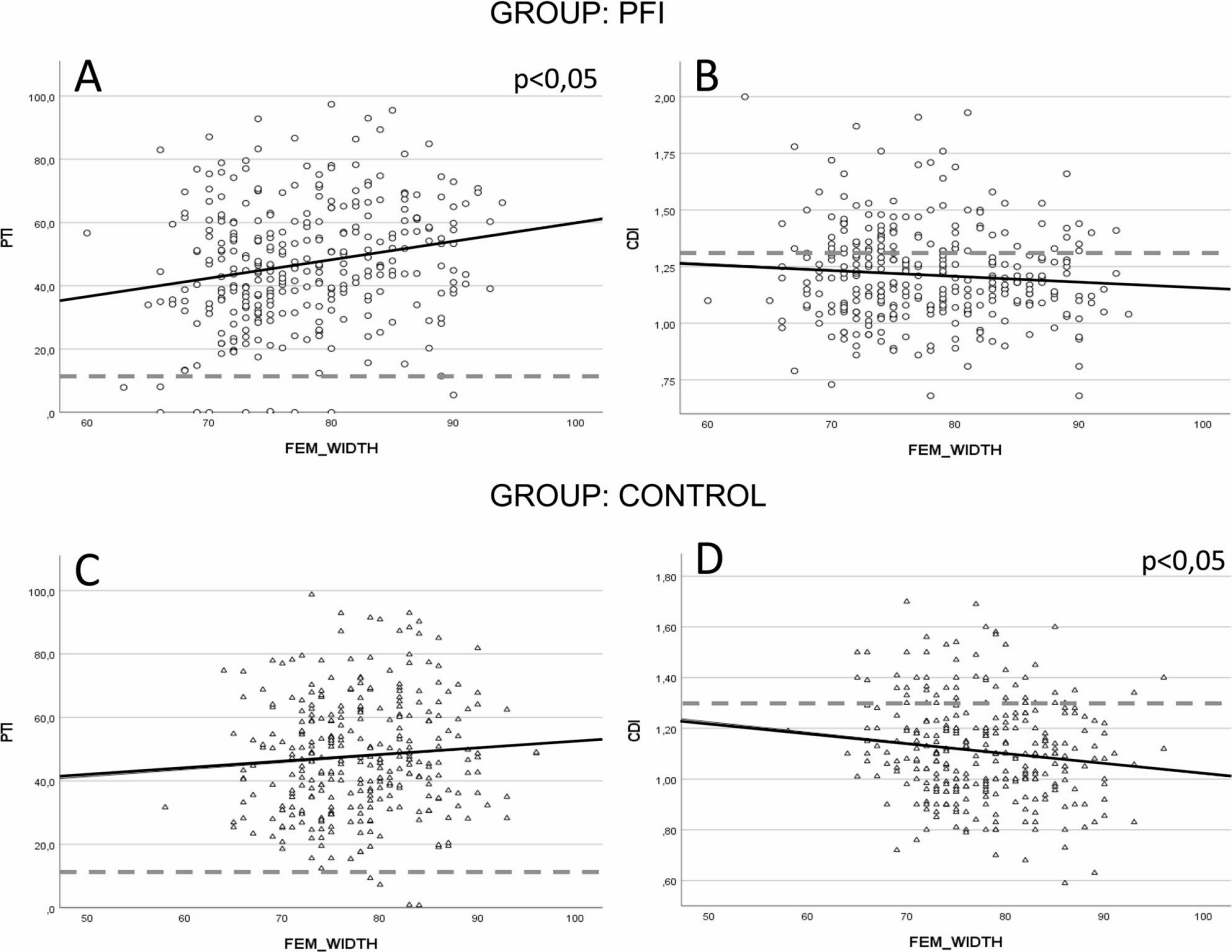
Illustrates the changes of patellar height, represented by the Patellotrochlear Index [PTI] (**A**, **C**) and the Caton-Dechamps index [CDI] (**B**, **D**) in relation to femoral width for both the PFI and control groups. The dashed lines are marking approximate cut offs for abnormal values in literature on adult PFI for PTI (<12,5) and CDI (>1.3)

PTI increased significantly in the PFI group by 5.9 (*p* < 0.001; R²=0.04) with larger femoral width, with no significant change in controls (Fig. 5A, C). The Caton-Deschamps Index (CDI) increased by 0.04 in controls (Fig. 5D), with a non-significant decreasing trend in the PFI group (Fig. 5B).

### Summary of statistical results

All changes associated with a 10 mm increase in femoral width, including p-values, standard errors, and R² values, are presented in Table 3. For improved clarity, parameters for which neither group showed a statistically significant association were omitted from the table.

**Table 3:** Change in morphometric parameters per 10 mm increase in femoral width

Measurement	PFI (Change ± SE)	β	CI 95%	CONTROL (Change ± SE)	β	CI 95%
TD (mm)	0.4 (± 0.1)	0.167	0.13–0.62	0.7 (± 0.1)	0.269	0.41–0.94
LTIA (°)	n.s.	n.s.	n.s.	1 (± 0.5)	0.112	0.02–2
FULK (°)	n.s.	n.s.	n.s.	3.5 (± 0.9)	0.225	1.8–5.2
TILT (°)	-1.9 (± 0.8)	-0.139	-3.5 - -0.4	-3.4 (± 0.8)	-0.227	-5.1 - -1.8
TTPCL (mm)	1.2 (± 0.5)	0.138	0.24–2.16	n.s.	n.s.	n.s.
PTI	5.9 (± 1.6)	0.205	2.75–8.99	n.s.	n.s.	n.s.
CDI	n.s.	n.s.	n.s.	0.04 (± 0.02)	-0.139	-0.07 - -0.01

Presents the results of the regression analysis (Change ± Standard Error [SE], regression coefficients [β] and 95% confidence intervals [CI 95%]). All statistically significant changes (*p* < 0.05) are related to an increase of 10 mm bicondylar femoral width with the femoral width as independent parameter for knee size. Parameters not reaching statistical significance are marked with n.s. (not significant)

*PFI *Patellofemoral instability, *CONTROL *Control group, *SE *Standard error, *TD *Trochlea depth, *LTIA *Lateral trochlea inclination angle, *TSA *Trochlea sulcus angle, *LAUR *Angle of Laurin, *FULK *Angle of Fulkerson, *TILT *Patellar inclination angle, *TTTG *Tibial tubercle to trochlea groove distance, *TTPCL *Tibial tubercle to posterior cruciate ligament distance, *PTI *Patellotrochlear-Index, *CDI *Caton-Dechamps-Index

*X = the change of the parameter reached no statistical significance (*p* > 0.05)

## Discussion

This study aimed to determine whether anatomic risk factors for atraumatic LPD change with increasing knee size and whether these differences vary between patients with and without a history of patellar dislocation. Since body height varies significantly among skeletally immature patients, knee size does not directly correlate with age [5, 17–19]. Our data support this, showing only a weak or no correlation between knee size and age.

In a group with first-time lateral patellar dislocation and a normally developing control group, femoral width was used to individualize measurements in growing patients due to its strong correlation with body height [20–22]. A statistically significant difference (*p* < 0.05) was found between the PFI and control groups for all measured parameters except PTI. These findings align with existing literature on risk factors for patellofemoral instability [29].

Statistically significant changes were observed in TD, LTIA, the Angle of Fulkerson, patellar inclination angle, TTPCL, and patellar height measurements (PTI and CDI) relative to individual knee size. This study therefore demonstrates that risk factors for first time atraumatic LPD manifest early in skeletal development and persist at a pathological level during during growth when comparing patients with a history of PFI to controls.

TD, LTIA, and TSA are key indicators of trochlear dysplasia [11, 30]. Lower TD and LTIA values correlate with a higher risk of non-traumatic LPD, while a higher TSA indicates a flatter trochlea. Our findings show a significant increase in trochlear depth in both groups. Furthermore, LTIA increased with femoral width only in the control group, remaining consistently low in LPD patients. This aligns with recent findings by Orellana et al. [31] as well as other previous studies suggesting that trochlear dysplasia evolves during maturation [17, 24, 28]. Factors like patella alta or lateralization may limit trochlear development if present early [3]. However, unlike previous research, we found no significant change in TSA relative to knee size, despite studies by Choi et al. [17], Düppe et al. [24], and Pruneski et al. [26] indicating a decrease over time. Richmond et al. [27] observed a similar trend in cadaveric knees, but their cohort (ages 2–8) was younger than ours. Causes excluding age differences in cohorts for this deviation from literature might be different measurement techniques (e.g. measurement on subchondral bone in our study and measurement at cartilage level by Choi et al. [17]), differences in sex ratio between cohorts as females may show differing development compared to male individuals [32].

Patellar tilt, a key factor in tracking and stability, showed significant differences between the PFI and control groups based on the patellar inclination angle. The PFI group had a slower reduction in patellar tilt, while healthy controls improved more rapidly, aligning with Düppe et al. [24]. These differences highlight the need for age- or knee size-specific assessments in evaluating patellar alignment and instability risk. Notably, the Fulkerson angle significantly increased, and the Angle of Laurin trended upward (*p* = 0.052) in healthy controls, whereas values remained stable in the PFI group. This contrasts with Düppe et al. [24], who found no significant age-related changes but observed a near-significant p-value of 0.056 for the Fulkerson angle.

In terms of lateralization measurements, our study revealed significant differences only in the PFI group for tibial tuberosity to posterior cruciate ligament distance (TTPCL). The increase in TTPCL distance with femoral width in the PFI group aligns with current literature, where TTTG or TTPCL was correlated with age, with Dickens et al. [33] noting the most pronounced changes in younger patients aged 1 to 9 years and lesser changes in adolescents over 10 years of age [17, 34]. However, TTTG did not change with knee size in either group, despite Pennock et al. [25] demonstrating a correlation between TTTG and increasing body height. While TTTG is widely employed to assess patellar lateralization, caution is advised as it is measured across the knee joint; femoral rotation can therefore significantly impact the measurement if standardized procedures are not followed during MRI acquisition. This can potentially lead to discrepancies when comparing it to CT results or results between different hospitals or institutes [35]. Moreover, Dong et al. [36] found that in cases of mild to high-grade trochlear dysplasia, interrater reliability for TTTG distance decreases significantly, suggesting that TTPCL distance may be more specific to tubercle lateralization as it does not pertain to the dysplastic trochlear groove.

Patellar height was assessed using the Patellotrochlear Index (PTI) and the Caton-Deschamps Index (CDI), revealing knee size-related variations (Fig. 5A-D). In the PFI group, PTI increased significantly with greater femoral width, while no significant changes were observed in the control group. Conversely, the CDI showed significant differences in the control group, with a trend toward significance in the PFI group, though statistical significance was not reached (*p* = 0.132). These findings align with previous research by Pruneski et al. [26] and Düppe et al. [24], which examined CDI changes in relation to patient age for both PFI and control groups. The observed decrease in patellar height in the healthy control group may support the normal development of the trochlear groove [3]. Additionally, the differences in CDI between the two groups highlight the importance of individualized assessments in paediatric populations, a need emphasized by Richmond et al. [27] in their analysis of normal patellar development in children.

This study has several limitations, primarily its retrospective design, which restricted the availability of relevant clinical, demographic, and maturational variables. Data on height, weight, body mass index, generalized joint hypermobility, duration of symptoms, family history, habitual or sport-specific activity level, and pubertal developmental stage were not consistently or systematically documented in the medical records and therefore could not be included in the analyses. As abnormal BMI, higher training loads, and pubertal maturation may influence both the risk of patellofemoral instability and skeletal growth, the observed associations between femoral width and anatomical parameters should be interpreted with caution [37, 38]. Future prospective studies incorporating these variables may allow for better-adjusted and developmentally stratified analyses and may provide additional insight into the relationship between knee morphology and patellofemoral instability during skeletal development.

Second, standardized clinical outcome data were not available for the study cohort. Because this was a retrospective MRI-based investigation, information on symptom severity, functional impairment, subjective instability, and validated clinical outcome scores was not consistently documented. As a result, the association between radiological parameters and clinical severity of patellofemoral instability could not be assessed directly in this cohort. Although imaging findings do not always correspond to symptoms or functional limitation in individual patients, previous studies have demonstrated associations between anatomical risk factors and clinically relevant instability outcomes, particularly recurrent dislocation [8, 16, 39]. Future prospective studies combining radiological measurements with standardized clinical and patient-reported outcome data are therefore needed.

Third, the retrospective imaging-based design did not allow for systematic longitudinal follow-up of the cohort. As a result, the progression of symptoms, recurrence of instability, or long-term functional outcomes following the initial lateral patellar dislocation could not be evaluated. Longitudinal studies incorporating follow-up assessments would be valuable to better understand how anatomical risk factors relate to persistent instability or disability.

Additionally, the inclusion period differed between cohorts, as the PFI cohort was collected over a longer time frame due to lower annual case numbers of first-time patellar dislocation. However, all MRI examinations were performed using standardized imaging protocols and positioning, and both groups were matched by age and sex. Therefore, we consider the risk of temporal selection bias to be limited.

Furthermore, the study included relatively few patients younger than eight years of age, which may limit generalizability across the entire spectrum of skeletal development. Although the cohorts were sex-matched, sex-specific developmental differences in patellofemoral morphology may exist. A separate sex-stratified analysis was conducted as part of an independent investigation [32].

Finally, the single-center study design may limit the generalizability of the findings to other populations. While the present study focused on morphological risk factors assessed by MRI, it did not include functional or biomechanical assessments that may further contribute to patellofemoral instability.

Patellofemoral instability (PFI) is a significant clinical concern, particularly among adolescents, due to the dynamic nature of knee anatomy during skeletal growth, which complicates diagnosis and treatment. Anatomic predisposition is a key factor in PFI risk [5]. The present results describe developmental patterns and group differences normalized to knee size, but do not determine clinical diagnostic thresholds. Future studies with prospective design and ROC analysis are required to define normal and abnormal thresholds. Our findings align with existing literature on anatomical risk factors for patellar instability and joint morphology during growth [17, 24–28]. Experts increasingly advocate for individualized treatment approaches, and the differences observed between the PFI and control groups highlight the importance of personalized assessments, particularly in growing patients [23]. Specifically, parameters that are already out of range at baseline and show a tendency to become more pathological with increasing femoral width should be regarded as “high-alert” variables. In such cases, progressive deviation from normal morphology in a growing knee may indicate an unfavourable developmental trajectory and could, in selected patients, serve as an early signal to consider preventive or conservative measures (e.g. patellofemoral-focused physiotherapy, sport modification). Preventive programs have already been shown to reduce ACL injury risk in high-risk athletes [30]. This might emphasize the need for establishing such preventive programs in patients at risk for first-time lateral patella dislocation (e.g. females in high-risk sports) [6]. Hingelbaum et al. [40] and Uimonen et al. [15] underscore the importance of further investigation into these morphometric changes. Enhanced understanding in this area may enable more precise surgical planning and optimized rehabilitation protocols, ultimately improving patient outcomes.

This study provides reference data on the developmental behaviour of patellofemoral risk factors normalized to knee size in skeletally immature patients. However, the findings should be interpreted as descriptive and hypothesis-generating rather than as a basis for immediate clinical recommendations. Longitudinal prospective studies are needed to clarify how these parameters relate to knee development and the course of patellofemoral instability over time. In addition, future studies should determine whether measures such as trochlear dysplasia or patellar inclination may help establish diagnostic thresholds or support individualized treatment strategies in skeletally immature patients once validated in prospective cohorts.

## Supplementary Information

Additional file 1: Supplementary figures.

## Data Availability

The data will be made available upon request by the first author.
